# Serum ACSL4 levels in patients with ST-segment elevation myocardial infarction (STEMI) and its association with one-year major adverse cardiovascular events (MACE): A prospective cohort study

**DOI:** 10.1097/MD.0000000000036870

**Published:** 2024-01-12

**Authors:** Yun Hu, Qingye Li, Yinglin Wang

**Affiliations:** a Emergency Department, Wuhan Dongxihu District People’s Hospital, Wuhan, China; b Emergency Department, Hubei Provincial Hospital of Traditional Chinese Medicine, Wuhan, China.

**Keywords:** ACSL4, cytokines, ferroptosis, prognosis, STEMI

## Abstract

In the present prospective cohort research, we aimed to explore the serum levels of Acyl-CoA synthetase long-chain family member 4 (ACSL4) in patients with ST-segment elevation myocardial infarction (STEMI) and its association with 1-year major adverse cardiovascular events (MACE). This prospective cohort study recruited 507 patients who underwent percutaneous coronary intervention for the treatment of STEMI at our hospital during August 2019 to July 2022. The serum ACSL4, tumor necrosis factor-α, interleukin (IL)-6, IL-1β, and C-reactive protein levels were measured by enzyme-linked immunosorbent assay. Demographic and clinical statistics were also collected. In addition, all patients were followed up for 1 year, and patients with MACE were defined as poor prognosis group. All data used SPSS 26.0 to statistical analyses. The poor prognosis group had significantly higher age and low-density leptin cholesterol (LDLC) levels compared to the favorable prognosis group (*P* < .05). STEMI patients exhibited significantly elevated serum levels of ACSL4, tumor necrosis factor-α, IL-6, IL-1β, and C-reactive protein (*P* < .05). Serum ACSL4 and IL-1β levels in the poor prognosis group were remarkably enhanced compared to the favorable prognosis group. Curvilinear regression analysis demonstrated that ACSL4 was associated with LDLC and IL-1β. Moreover, ACSL4 (*B* = 0.138, 95% CI 1.108–1.189, *P* < .001), LDLC (*B* = 2.317, 95% CI 5.253–19.603, *P* < .001), and IL-1β (*B* = 0.061, 95%CI 1.008–1.122, *P* = .025) levels were the risk factors for STEMI patients with 1-year MACE. This study showed that the serum ACSL4 levels was remarkably elevated in STEMI patients. This study might provide new targets and a comprehensive approach to cardiovascular protection in STEMI patients.

## 1. Introduction

Acute myocardial infarction (AMI) is a dangerous cardiac event in patients with coronary heart disease.^[1]^ It is generally believed that the main cause of AMI is the changes in atherosclerotic plaques in the patient’s body, such as plaque rupture or ulceration, leading to secondary thrombus formation, resulting in complete interruption of coronary artery blood flow or the formation of a syndrome due to extreme reduction in blood flow.^[2,3]^ Based on changes in the electrocardiogram, AMI is divided into ST-segment elevation myocardial infarction (STEMI) and non-ST-segment elevation myocardial infarction.^[4]^ The reported incidence rate of STEMI ranges from 77 to 121 cases per 100,000 people.^[5]^ patients require urgent medical intervention, usually through percutaneous coronary intervention (PCI) to restore blood flow and reduce myocardial damage.^[6]^ Compared to non-ST-segment elevation myocardial infarction patients, STEMI patients have a higher short-term mortality rate and poorer long-term prognosis after PCI.^[7,8]^ Therefore, comprehensive assessment and identification of STEMI patients at risk of poor prognosis are necessary for further intervention and care.

There are many mechanisms that contribute to ischemia-reperfusion injury, including inflammatory response, oxidative stress, cell apoptosis, and ferroptosis.^[9–11]^ Among them, ferroptosis is a newly discovered form of cell death in recent years.^[12]^ Studies have shown that inhibiting ferroptosis-induced cardiomyocyte death can alleviate myocardial ischemia-reperfusion injury.^[13,14]^ In a recent review article published in 2022, the authors also discussed the significant detrimental role of ferroptosis in the progression of ischemia-reperfusion and suggested that targeting the ferroptosis pathway could be a promising approach for treating ischemia-reperfusion injury.^[15]^ These findings indicate that the ferroptosis pathway may contribute to myocardial ischemia-reperfusion injury. Acyl-CoA synthetase long-chain family member 4 (ACSL4) is a key enzyme involved in regulating lipid composition and has been shown to be implicated in ferroptosis.^[16]^ Evidence suggests that preventing myocardial ischemia/reperfusion injury can be achieved by inhibiting the ACSL4-mediated ferroptosis pathway.^[17]^ This indicates that ACSL4 plays a role in ferroptosis and ischemia-reperfusion injury. However, there is currently no clinical study on the impact of ACSL4 expression in STEMI patients on patient prognosis.

In the present prospective cohort research, we aimed to explore the serum ACSL4 levels in patients with STEMI and its association with 1-year major adverse cardiovascular events (MACE). This study might reveal the clinical significance of ACSL4 in STEMI patients, as well as provide novel research targets for STEMI treatment.

## 2. Methods

### 2.1. Research design

This study was a prospective cohort study and recruited 507 patients who underwent PCI for the treatment of STEMI at our hospital during August 2019 to July 2022. In addition, we collected serum samples from 150 healthy volunteers as controls. The study was approved by our hospital ethics committee. All subjects agreed to participate in this study and signed an informed consent form.

### 2.2. Inclusion and exclusion criteria

STEMI is characterized by characteristic symptoms of myocardial ischemia, persistent ST-segment elevation on electrocardiogram (ECG), and subsequent release of biomarkers indicating myocardial necrosis.^[18]^ The specific diagnostic criteria are as follows^[19,20]^: cardiac biomarkers (preferably cTn) > 0.1 ng/mL (upper normal limit) or rising and falling, along with evidence of at least one of the following signs of myocardial ischemia and necrosis: (1) clinical symptoms of myocardial ischemia (symptoms lasting ≥ 20 min); (2) new ST-segment elevation ≥ 0.2 mV in leads V2 and V3 (≥0.25 mV in males < 40 years, ≥0.15 mV in females), or ≥0.1 mV in other leads and limb leads; (3) pathological Q waves; (4) imaging evidence of new loss of myocardial viability or regional wall motion abnormalities. Exclusion criteria include: (1) patients with a history of myocardial infarction; (2) patients who received treatment more than 12 hours after symptom onset; (3) patients with stable angina, myocardial bridging, coronary artery anomalies, cardiomyopathy, valvular heart disease, congenital heart disease, or heart failure; (4) patients who received steroids, anti-inflammatory drugs, antibiotics, nonsteroidal anti-inflammatory drugs, immunesuppressants, etc within 1 month; (5) patients with rheumatic diseases, hematological diseases; (6) patients with severe infections, severe liver or kidney disease, malignancies, or cardiovascular dysfunction.

### 2.3. Research variables

Baseline characteristics including age, Body Mass Index, sex, diastolic blood pressure, systolic blood pressure, comorbidities (hypertension, diabetes, atrial fibrillation), numbers of diseased vessels were collected. Routing whole blood test was performed using an automatic biochemical analyzer (Hitachi 7600, Hitachi Corporation, Japan) and the levels of fasting plasma glucose (FPG), total cholesterol, triglyceride, high-density lipoprotein cholesterol, low-density lipoprotein cholesterol (LDLC), cardiac troponin I (cTnI), D-dimer, creatinine, N-terminal proBNP were recorded. In addition, all patients were followed up for 1 year, and patients with one of the following conditions during follow-up: cardiac death, heart failure, cardiogenic shock, recurrent myocardial infarction, arrhythmia with hemodynamic disturbances defined as MACE (poor prognosis group).

### 2.4. Blood sampling measurement

The serum ACSL4, tumor necrosis factor-alpha (TNF-α), interleukin (IL)-6, IL-1β and C-reactive protein (CRP) levels were measured by enzyme-linked immunosorbent assay (ELISA). Blood samples of fasting cubital venous (5 mL) were collected within 24 hours after admission for all cases. Samples were centrifuged at 2000 g for 15 minutes, following with ELISA tested using commercially available kits (ACSL4 MBS9331516 MyBioSource, TNF-α MBS824943 MyBioSource, IL-6 MBS175877 MyBioSource, CRP MBS177184 MyBioSource, IL-1β MBS175901 MyBioSource).

### 2.5. Sample size

${\left(Z_{1-\alpha /2}*\sqrt{p*\left(1-p\right)}\right)}^{2}/\delta^{2}$ was used to calculate the sample size. Z_1-α/2_ = 1.96, δ = 0.04, *p* represents the sensitivity, which in this study is expected to be 70%. Thus, both groups had a minimum sample size of 504 participants.

### 2.6. Statistical analysis

The normal distribution of data was confirmed by Kolmogorov-Smirnov analysis. Normal distribution data were expressed by mean (SD) while non-normal distribution data median (range). Mann-Whitney test was used for comparison between 2 groups. Chi square test was used for rates. Curvilinear regression analysis was used for correlation analysis. The role of serum ACSL4 in the prediction of patient prognosis was analyzed using receiver operating characteristic curve analysis. Logistic regression was performed for risk factors of poor prognosis. *P* < .05 regarded significant difference. All data used SPSS 26.0 to statistical analyses.

## 3. Results

### 3.1. Clinical characteristics of all participants

This prospective cohort study included 507 STEMI patients who underwent PCI treatment at our hospital. All patients were followed up for 1 year after treatment and were divided into a favorable prognosis group (n = 363) and a poor prognosis group (n = 144) based on the occurrence of MACE. The demographic and clinical data of the 2 groups were compared, as shown in Table 1. The results showed that the poor prognosis group had significantly higher age and LDLC levels compared to the favorable prognosis group (*P* < .05). Additionally, there were no significant differences between the 2 groups in terms of sex, body mass index, systolic blood pressure, diastolic blood pressure, total cholesterol, triglyceride, high-density lipoprotein cholesterol, cardiac troponin I, D-dimer, creatinine, and N-terminal proBNP.

**Table 1:** Demographic and clinical data of all subjects.

Variable	Favorable prognosis, n = 363	Poor prognosis, n = 144	*P* value
Age, y	49 (31–67)	51 (33–72)	.047
Sex, female (%)	188 (51.8)	69 (48.0)	.431
BMI	26.11 (20.02–31.62)	26.49 (19.96–31.60)	.425
SBP (mm Hg)	130.41 (103.14–155.61)	133.76 (103.03–155.61)	.156
DBP (mm Hg)	86.43 (67.00–105.09)	86.77 (67.53–104.84)	.787
TC (mmol/L)	3.70 (2.47–4.66)	3.80 (2.47–4.69)	.551
TG (mmol/L)	1.35 (1.02–1.63)	1.33 (1.03–1.61)	.366
HDLC (mmol/L)	1.10 (0.93–1.26)	1.12 (0.93–1.26)	.154
LDLC (mmol/L)	2.30 (1.50–3.07)	2.66 (1.94–3.23)	<.001
cTnI (ng/L)	15.80 (7.22–22.20)	16.08 (7.25–22.22)	.265
D-D (μg/mL)	0.96 (0.41–1.41)	0.99 (0.43–1.40)	.636
Cr (μmol/L)	72.85 (45.98–96.63)	75.43 (46.85–95.66)	.341
NT-proBNP (pg/mL)	619.61 (262.11–909.53)	607.21 (254.26–908.06)	.533

BMI = body mass index, Cr = creatinine, cTnI = cardiac troponin I, DBP = diastolic blood pressure, D-D = D-dimer, HDLC = high-density leptin cholesterol, LDLC = low-density leptin cholesterol, NT-proBNP = N-terminal proBNP, SBP = systolic blood pressure, TC = total cholesterol, TG = triglyceride.

### 3.2. Serum levels of ACSL4 and inflammatory factors in STEMI patients

**Figure 1.**
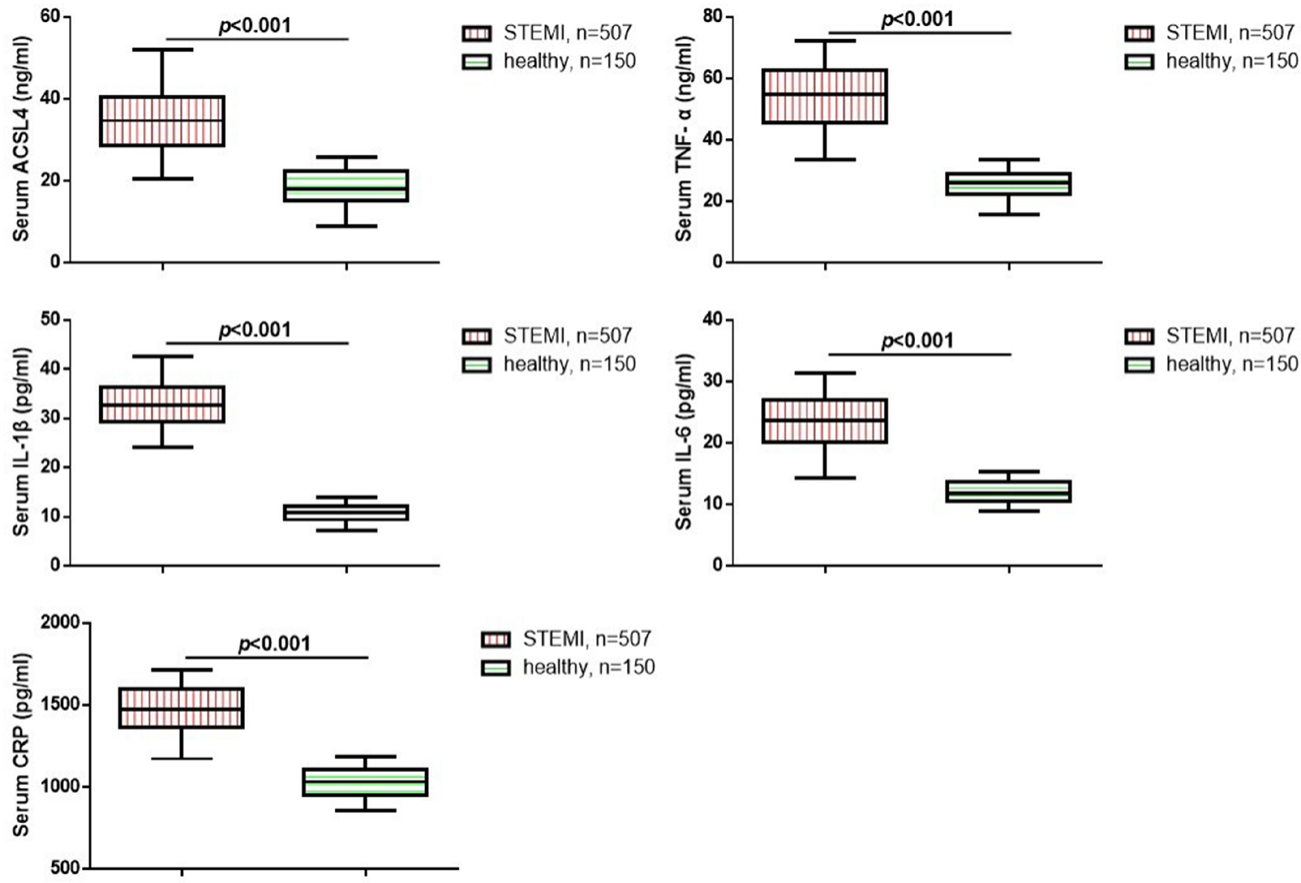
Comparisons of ACSL4 and other serum biomarkers in all subjects. ACSL4 = acyl-CoA synthetase long-chain family member 4.

**Figure 2.**
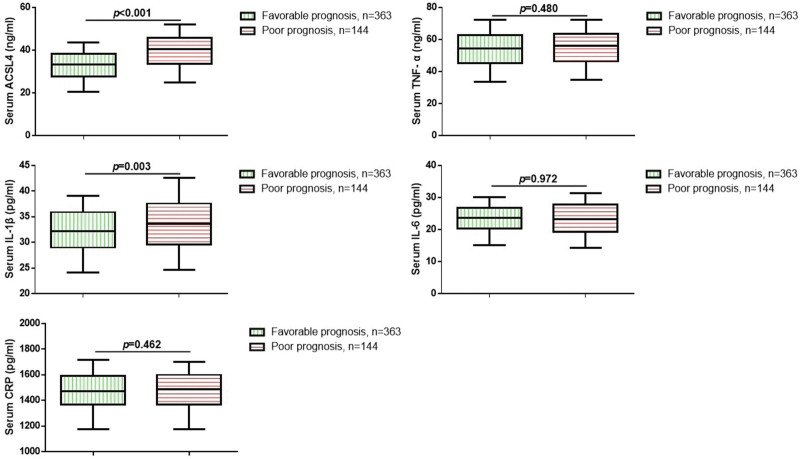
Correlation of ACSL4, cytokines, and prognosis in all STEMI patients. ACSL4 = acyl-CoA synthetase long-chain family member 4, STEMI = ST-segment elevation myocardial infarction.

Further, we investigated the serum levels of ACSL4 and inflammatory cytokines in STEMI patients. We measured the serum levels of ACSL4, TNF-α, IL-6, IL-1β, and CRP in of all STEMI patients and 150 healthy volunteers using the ELISA method. As shown in Figure 1, compared to the healthy population, STEMI patients exhibited significantly elevated serum levels of ACSL4, TNF-α, IL-6, IL-1β, and CRP (*P* < .05). We attempted to further explore the association between these serum biomarkers and the prognosis of STEMI patients. The results suggested a significant increase in serum ACSL4 and IL-1β levels in the poor prognosis group compared to the favorable prognosis group (Fig. 2, *P* < .05). There was no significant difference in TNF-α, IL-6, and CRP levels between the 2 groups.

**Figure 3.**
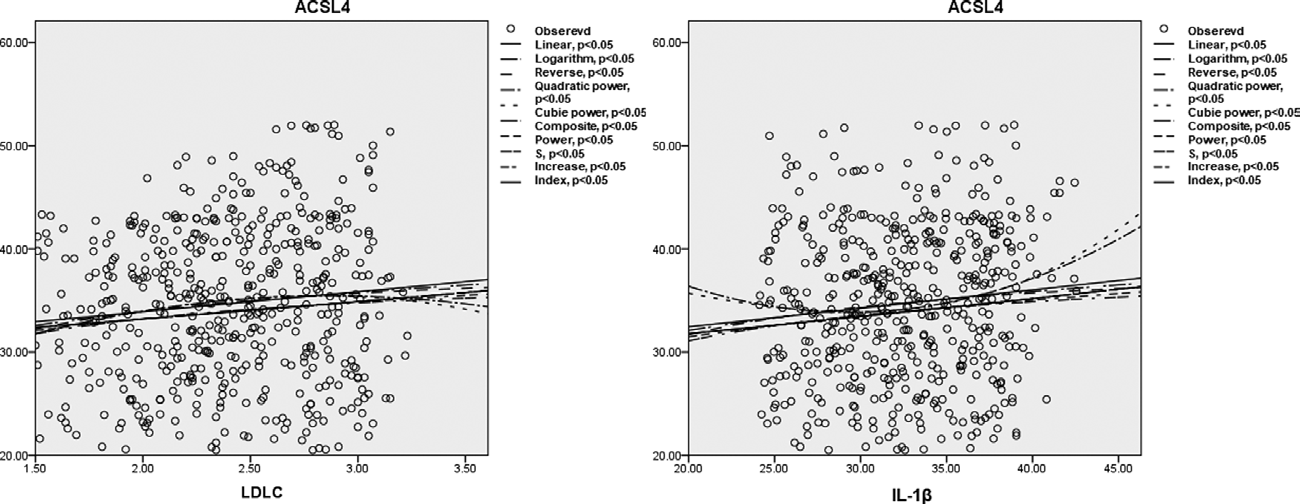
Curvilinear regression analysis of ACSL4 and serum biomarkers. ACSL4 = acyl-CoA synthetase long-chain family member 4.

We performed curvilinear regression analysis to examine the relationship between serum ACSL4 levels and inflammatory factors, as well as clinical factors. The results, as shown in Figure 3, demonstrated that ACSL4 was associated with LDLC and IL-1β.

### 3.3. ACSL4 for the prognosis in STEMI patients

**Figure 4.**
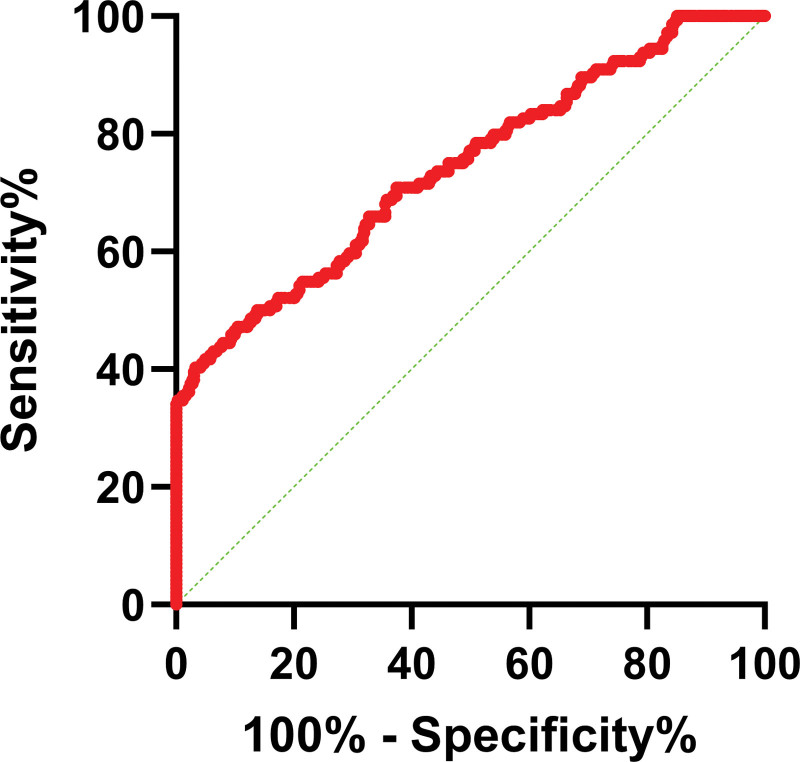
ROC curves for ACSL4 for the prognosis in STEMI patients. ACSL4 = acyl-CoA synthetase long-chain family member 4; ROC = receiver operating characteristic; STEMI = ST-segment elevation myocardial infarction.

We draw receiver operating characteristic curves to assess the role of ACSL4 for the prognosis in STEMI patients. The result showed that ACSL4 could be a potential predictive biomarker in predicting poor prognosis in STEMI patients, the AUC was 0.742, with a cutoff value of 35.78 ng/mL, sensitivity of 68.8%, and specificity of 64.5% (Fig. 4).

### 3.4. Risk factors of STEMI patients with 1-year MACE by logistic regression analysis

Finally, we used entry method for logistic regression to analyze the risk factors of STEMI patients with 1-year MACE. Firstly, we performed univariate logistic regression analysis and found that Age, ACSL4, LDLC, and IL-1β levels were risk factors for adverse prognosis in STEMI patients (Table 2). Subsequently, we conducted multivariate logistic regression analysis with the risk factors for adverse prognosis identified in the univariate analysis as covariates, and the results showed that ACSL4 (*B* = 0.138, 95% CI 1.108–1.189, *P* < .001), LDLC (*B* = 2.317, 95% CI 5.253–19.603, *P* < .001), and IL-1β (*B* = 0.061, 95% CI 1.008–1.122, *P* = .025) levels were the risk factors for STEMI patients with 1-year MACE.

**Table 2:** Logistic regression for risk factors of STEMI patients with poor prognosis.

Variables	Odds ratio	95% CI	*P* value
Univariate			
Age	1.021	1.002–1.039	.027
Sex	0.856	0.582–1.260	.432
BMI	1.025	0.964–1.089	.432
SBP	1.009	0.996–1.024	.183
DBP	1.003	0.984–1002	.786
TC	1.125	0.808–1.567	.485
TG	0.656	0.195–2.206	.496
HDLC	4.649	0.554–38.990	.157
LDLC	8.524	4.811–15.104	<.001
cTnI	1.027	0.979–1.076	.276
D-D	1.169	0.573–2.383	.668
Cr	1.007	0.993–1.021	.317
NT-proBNP	1.000	0.999–1.001	.507
ACSL4	1.151	1.113–1.189	<.001
TNF-α	1.006	0.988–1.025	.498
IL-1β	1.007	1.028–1.127	.002
IL-6	0.999	0.954–1.046	.968
CRP	1.000	0.999–1.002	.579
Multivariable			
Age	1.018	0.996–1.040	.103
LDLC	10.147	5.253–19.603	<.001
ACSL4	1.148	1.108–1.189	<.001
IL-1β	1.063	1.008–1.122	.025

ACSL4 = acyl-CoA synthetase long-chain family member 4, BMI = body mass index, Cr = creatinine, cTnI = cardiac troponin I, CRP = C-reactive protein, DBP = diastolic blood pressure, D-D = D-dimer, HDLC = high-density leptin cholesterol, IL = interleukin, LDLC = low-density leptin cholesterol, NT-proBNP = N-terminal proBNP, SBP = systolic blood pressure, STEMI = ST-segment elevation myocardial infarction, TC = total cholesterol, TG = triglyceride, TNF-α = tumor necrosis factor α.

## 4. Discussion

Myocardial infarction is a leading cause of morbidity and mortality worldwide, with over 15% of deaths attributed to heart attacks each year.^[21]^ Furthermore, study has found an increasing incidence of STEMI, which due to delays in diagnosis and management, leads to poor prognosis in patients.^[22]^ Therefore, there is an urgent need to develop novel biomarkers and comprehensive approaches for the assessment, early management, and intervention in STEMI patients to mitigate adverse outcomes. In this study, we identified serum level of ACSL4 was the risk factors for STEMI patients with 1-year MACE.

In recent years, several studies have focused on the abnormal expression of biomarkers in STEMI patients. Karagiannidis et al^[23]^ found that higher levels of ceramide were associated with larger thrombus volumes, indicating that quantification of serum ceramides may improve risk stratification in STEMI patients. Gu et al^[24]^ confirmed that serum FGF21 levels in STEMI patients can be used to predict the occurrence of MACE in emergency PCI patients. Mechtouff et al^[25]^ demonstrated that elevated levels of soluble form suppression of tumorigenicity 2 (sST2) in serum 24 hours after admission are associated with an increased risk of adverse clinical events in STEMI patients. Additionally, Groot et al^[26]^ suggested that higher levels of IL-6 at 24 hours after STEMI onset were associated with larger infarct sizes and decreased cardiac function at 4 months. In our study, we found significantly higher levels of inflammatory cytokines TNF-α, IL-6, IL-1β, and CRP in the serum of STEMI patients compared to healthy individuals.

ACSL4 is involved in intracellular fatty acid metabolism and signaling, and its expression and molecular mechanisms have attracted extensive attention in various diseases. In lung cancer, lower ACSL4 levels may indicate poor clinical outcomes and prognosis.^[27]^ In hepatocellular carcinoma, ACSL4 is crucial for sorafenib-induced ferroptosis and can be used to predict hepatocellular carcinoma sensitivity to sorafenib.^[28]^ This may be related to the activation of ACSL4 pathway inducing cellular ferroptosis. Additionally, ACSL4 is closely associated with neurodegenerative diseases such as Alzheimer disease^[29]^ and Parkinson disease,^[30]^ and increased ACSL4 expression may lead to oxidative stress, apoptosis, and ferroptosis of neuronal cells, thereby impairing the function of the nervous system.^[31]^ In the study of ischemia-reperfusion injury, Feng et al found that ACSL4 plays a critical role in the process of ischemia-reperfusion injury, and inhibiting ACSL4 before reperfusion can prevent the activation of the ferroptosis pathway and cell death.^[32]^ Fan et al also confirmed that baicalin can prevent myocardial ischemia-reperfusion injury by inhibiting the ACSL4-linked ferroptosis pathway.^[17]^ Therefore, we measured the serum ACSL4 levels in all study subjects and found that the serum ACSL4 levels were significantly increased in STEMI patients compared to healthy controls. Additionally, we also found ACSL4 levels were correlated with LDLC and IL-1β, which is consistent with the findings of Tao et al,^[33]^ who also observed increased ACSL4 expression in ischemia-reperfusion injury and demonstrated that inhibiting ACSL4 can reduce the expression of inflammatory factors such as IL-6 and TNF-α. Furthermore, our study results indicated that serum level of ACSL4 was the risk factors for STEMI patients with 1-year MACE.

The current study has several limitations that merit consideration. Firstly, the sample size was relatively small, which could affect the generalizability of the findings. Secondly, our analysis only assessed a limited number of inflammatory factors, which may have excluded other potentially relevant variables. Lastly, further in-depth research is needed to elucidate the molecular mechanisms by which ACSL4 is involved in the development of STEMI. In the future, more experiments will need to be designed to investigate this.

## 5. Conclusion

This study showed that the serum ACSL4 levels was remarkably elevated in STEMI patients. In addition, serum level of ACSL4 was the risk factors for STEMI patients with 1-year MACE. This study might provide new targets and a comprehensive approach to cardiovascular protection in STEMI patients.

## Author contributions

**Data curation:** Yun Hu, Yinglin Wang.

**Investigation:** Yun Hu.

**Methodology:** Yinglin Wang.

**Project administration:** Qingye Li.

**Software:** Yinglin Wang.

**Supervision:** Qingye Li.

**Writing – review & editing:** Qingye Li.

**Writing – original draft:** Yun Hu.
